# RAW264.7 Macrophages as a Polarization Model in the Context of Pancreatic Cancer and Chemokine Release

**DOI:** 10.3390/biology14040320

**Published:** 2025-03-21

**Authors:** Aydar Khabipov, Lea Miebach, Maik Lenz, Stephan Kersting, Sander Bekeschus

**Affiliations:** 1 Department of General, Visceral, Thoracic, and Vascular Surgery, Greifswald University Medical Center, Ferdinand-Sauerbruch-Str., 17475 Greifswald, Germany; 2 ZIK Plasmatis, Leibniz Institute for Plasma Science and Technology (INP), Felix-Hausdorff-Str. 2, 17489 Greifswald, Germany; 3 Department of Dermatology and Venerology, Rostock University Medical Center, Strempelstr. 13, 18057 Rostock, Germany

**Keywords:** CCR2, CCR4, macrophage polarization, TME, tumor microenvironment

## Abstract

The TME is a critical niche for determining the fate of cancer therapy. Tumor cells often polarize nontumor cells, including immune cells, in the TME to favor cancer growth. In pancreatic cancer, macrophages are associated with poor therapy outcomes and unfavorable survival, especially when rendered into M2 macrophages. The latter show features also found in so-called tumor-associated macrophages (TAM), which are described as protecting and propelling tumor growth. In this context, it has been understudied which pancreatic cancer chemokines contribute to macrophage polarization. To this end, we analyzed murine RAW264.7 macrophages and Panc02 and PDA6606 pancreatic cancer cells in mono- and coculture to identify release patterns of 13 chemokines. Artificial macrophage polarization confirmed prominent changes in surface receptor and chemokine secretion profiles. Strikingly, RAW264.7 cocultures with Panc02 or PDA6606 were congruent in showing elevated levels of CCL2, CCL5, CCL17, CCL20, CCL22, CXCL5, and CXCL10. Further underlining the suitability of our in vitro model, both pancreatic cancer cell lines showed similar modulation of the critical macrophage polarization markers arginase, CD206, and iNOS, as well as chemokine receptors CCR2 and CCR4. Collectively, we demonstrated that our model is suitable for testing the roles and functions of chemokines in macrophage polarization by pancreatic cancer cells.

## Simple Summary

In solid tumors, cancer cells are surrounded by nontumor cells, such as immune and stromal cells. Together with non-cellular components, they form the tumor microenvironment (TME). Cancer cells can hijack nontumor cells in the TME to support tumor growth. Therefore, the TME has a significant influence on the success of cancer therapy. In pancreatic cancer, immune cells of the innate immune system, macrophages, have been shown to negatively impact the survival rate of cancer patients. However, the mediators (chemokines) released by pancreatic cancer cells to control macrophages are understudied. In the present study, we characterized interactions between a murine macrophage cell line and two pancreatic cancer cell lines in vitro. In screening 13 different chemokines, we observed a congruent increase in seven. This was paralleled by an increased expression of surface markers associated with a protumoral macrophage phenotype. Our findings are an essential basis for functional studies and might help to improve and develop novel therapies for pancreatic cancer in the future.

## 1. Introduction

The tumor microenvironment (TME) is critical for the efficacy of anticancer therapies [1]. The TME consists of several cellular and non-cellular components that can alter the activity of cells, the penetration of anticancer compounds, and tumor growth. For instance, it has been observed in pancreatic cancer that an immunosuppressive TME is associated with poor treatment outcomes and survival [2]. Many cell types can contribute to immunosuppressive microenvironments, including lymphocytes, fibroblasts, and myeloid cells, such as macrophages [3,4]. Therefore, it is critical to understand the delicate crosstalk between all cell types in the TME to understand if and how therapeutic approaches are necessary and possible to steer cellular polarization in favor of anticancer therapies and overall survival, especially in pancreatic cancer patients.

Pancreatic cancer is the 12th most common cancer globally, showing about 500,000 new cases and approximately the same number of death cases per year [5]. Pancreatic cancer has a high mortality rate due to its often late diagnosis, typically at an advanced stage, either metastatic or locally unresectable, leading to an overall survival of only about 10% in many countries [6]. Even more concerning, for the few patients who are eligible for surgical resection, disease recurrence rates are greater than 75% [7]. Risk factors for pancreatic cancer include age, smoking, and chronic conditions, such as pancreatitis, diabetes, and obesity. On the molecular level, more than half of pancreatic cancers harbor KRAS, TP53, CDKN2A, and SMAD4/DPC4 mutations, leading to continuous cell proliferation and dysregulated cell cycle control and genomic stability [8]. On the cellular level, cancer-associated fibroblasts (CAFs) and tumor-associated macrophages (TAMs) are described as drivers of pancreatic cancer drug insensitivity and pathogenesis [9,10].

TAMs are polarized cells with features of so-called M2-polarized macrophages [11]. Although the traditional scheme of classically activated M1 and alternatively activated M2 macrophages and their subtypes (M2a, M2b, M2c, etc.) has been increasingly questioned in recent years, the general notion of distinct macrophage behavior under differing environmental conditions remains valid [12]. In pancreatic cancer, TAMs are believed to be generated through elevated monocyte attraction in the TME, followed by their polarization into M2 macrophages with their typical pro-tumorigenic and immunosuppressive features. Cytokines, chemokines, and growth factors play critical roles in these processes. For instance, monocyte chemoattractant protein 1 (MCP1/CCL2) and RANTES (CCL5) support monocyte infiltration, while interleukins (IL) 4, 10, and 13, together with tumor growth factor (TGF) β, enable M2 polarization [11,13].

Although chemokines have been studied extensively in the context of macrophage polarization, their presence and roles in pancreatic cancer are not fully understood. To this end, we propose a straightforward in vitro coculture model for studying the presence of 13 different chemokines in experimental and pancreatic cancer-driven macrophage polarization. We found a strong alignment of effects with two independent pancreatic cancer cell lines, underlining the principal suitability of this approach for future studies.

## 2. Materials and Methods

### 2.1. Cell Culture

Murine pancreatic adenocarcinoma cells Panc02, murine PDA6606 cells, and murine macrophage cell line RAW264.7 were cultured in a Roswell Park Memorial Institute (RPMI) 1640 cell culture medium supplemented with 10% fetal bovine serum, 1% glutamine, and 1% penicillin–streptomycin (Sigma, Darmstadt, Germany) according to the supplier’s instructions. All the cells were kept in a specialized breeding incubator (Binder, Neckarsulm, Germany) at 37 °C, 5% CO_2_, and 95% humidity.

### 2.2. Macrophage Polarization

For macrophage polarization, 1 × 10^5^ RAW264.7 murine macrophages were seeded in 2 mL fully supplemented cell culture medium per well in 6-well plates (Sarstedt, Germany). After 24 h and 48 h, the cell culture medium was supplemented with 20 ng/mL M-CSF to induce macrophage differentiation (M1/M2). In addition, macrophage polarization was induced by adding (final concentration) 300 U/mL interferon (IFN) γ and 1 µg/mL lipopolysaccharide (LPS) for the induction of M1 macrophages, or 40 ng/mL interleukin (IL) 4 and 30 ng/mL IL-13 (all PeproTech, Hamburg Germany) for the induction of M2 macrophages, both after 48 h. Microscopic analysis was performed after 72 h, following cell detachment using an Accutase Cell Detachment Solution (BioLegend, Amsterdam, The Netherlands) and subsequent fixation using 4% paraformaldehyde (PFA; BioLegend, Amsterdam, The Netherlands) for flow cytometric analysis of surface marker expression profiles. Single-cell suspensions were stained with antibodies (conjugate) targeting Arginase (allophycocyanine, APC), CCR2 (brilliant violet 650, BV650), CCR4 (brilliant violet 421, BV421), CD11b (APC/Alexa-Fluor 780), CD206 (Alexa-Fluor 488, AF488), F4/80 (peridinin chlorophyll protein-Cyanine5.5, PerCP-Cy5.5), and iNOS (phycoerythrin, PE) (all BioLegend, Amsterdam, The Netherlands). Antibody concentrations were determined in the pilot experiments. After washing, cells were acquired, and fluorescence intensities were measured using a 6-laser flow cytometer (CytoFLEX LX; Beckman-Coulter, Krefeld, Germany).

**Figure 1:**
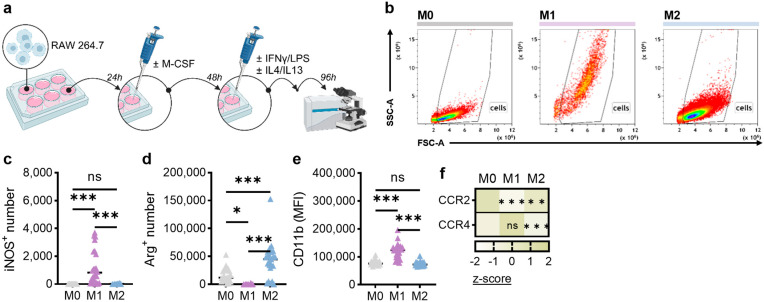
CCR2 and CCR4 expression on polarized macrophages in vitro. (**a**) Scheme of the experimental workflow; (**b**) representative flow cytometric dot plots; (**c**) quantification of iNOS^+^ macrophages (n = 3); (**d**) quantification of arginase (Arg)^+^ macrophages (n = 3); (**e**) CD11b expression (n = 3); (**f**) relative expression of C–C chemokine receptor type (CCR) 2 and CCR4 (n = 3). Statistical analysis was performed using one-way analysis of variance (ANOVA) (* *p* < 0.05; *** *p* < 0.001). Note: ns = non-significant.

### 2.3. Coculture Experiments

For the coculture experiments, 1.5 × 10^5^ Panc02 or PDA6606 pancreatic cancer cells were seeded in 2 mL of a fully supplemented cell culture medium per well in 6-well plates (Sarstedt, Germany). RAW264.7 murine macrophages were added at different effector-to-target ratios (ET (RAW264.7 macrophages: pancreatic cancer cells)) of 1:1, 1:3, and 1:5 after 24 h of incubation. Microscopic cluster analysis was performed after 48 h [14]. For the subsequent flow cytometric analysis of surface marker expression profiles on tumor-associated macrophages, the cells were detached after 72 h using an Accutase Cell Detachment Solution (BioLegend, Amsterdam, The Netherlands) and fixed using 4% PFA (BioLegend, Amsterdam, The Netherlands). Single-cell suspensions were stained with antibodies (conjugate) targeting Arginase (APC), CCR2 (BV650), CCR4 (BV421), CD11b (APC/Alexa-Fluor 780), CD206 (AF488), F4/80 (PerCP-Cy5.5), and iNOS (PE) (all BioLegend, Amsterdam, The Netherlands). Appropriate antibody concentrations were determined in the pilot experiments preceding coculturing. After washing, the cells were acquired using a 6-laser flow cytometer (CytoFLEX LX; Beckman-Coulter, Krefeld, Germany).

**Figure 2:**
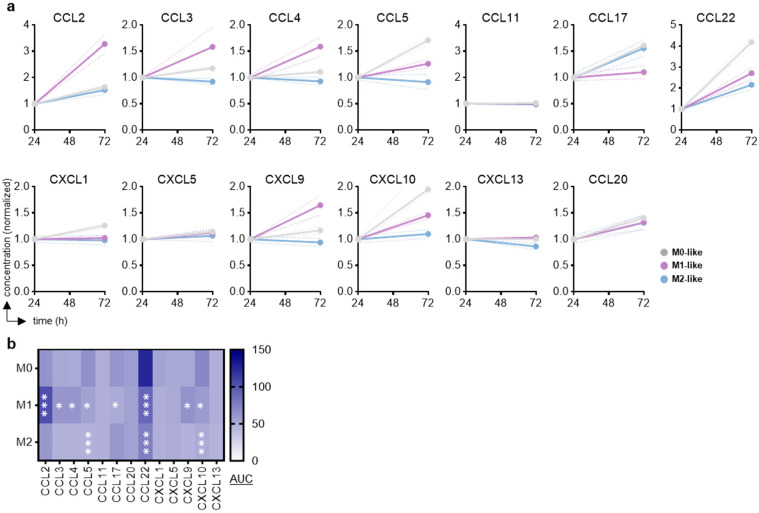
Chemokine release profiles in supernatants of polarized macrophages in vitro. (**a**) Normalized chemokine expression profiles and (**b**) a heat map showing their calculated areas under the curve (AUC; n = 3). Statistical analysis was performed using two-way analysis of variance (ANOVA) (* *p* < 0.05; *** *p* < 0.001).

### 2.4. Chemokine and Cytokine Analysis

Cell culture supernatants were sampled at 24 h, 48 h (coculture experiments only), and 72 h of incubation for the analysis of chemokine and cytokine profiling using a bead-based sandwich multi-analyte assay (LEGENDPlex MU Pro-inflammatory Chemokine Panel 1 (13plx); BioLegend, Amsterdam, The Netherlands) according to the manufacturer’s instructions. The predefined assay panel contained beads targeted against C–C motif chemokine ligand (CCL) 2 (monocyte chemotactic protein 1 (MCP1); ligand of CCR2), CCL17 (thymus- and activation-regulated chemokine (TARC)), CCL22 (macrophage-derived chemokine (MDC); both ligands of CCR4), and several other proinflammatory chemokines including CCL3 (macrophage inflammatory protein (MIP) 1α), CCL4 (MIP-1β), CCL5 (RANTES), CCL11 (eotaxin), CCL20 (MIP-3α), C–X–C motif chemokine ligand (CXCL) 1 (KC), CXCL5 (LPS-induced CXC chemokine (LIX)), CXCL9 (monokine induced by gamma interferon (MIG)), CXCL10 (interferon γ-induced protein 10 kDa (IP10)), and CXCL13 (B-lymphocyte chemoattractant (BLC)). The beads were labeled with fluorescent detection antibodies, and samples were acquired using flow cytometry (CytoFLEX LX; Beckman-Coulter, Krefeld, Germany). Absolute concentrations of the respective analytes were calculated against a standard curve using a specified cloud-based analysis software (version 2024.06.05; BioLegend, Amsterdam, The Netherlands).

### 2.5. Statistical Analysis

Graphing and statistical analysis were performed using Prism 9.5.1 (GraphPad Software, La Jolla, CA, USA) and one-way or two-way analysis of variance (ANOVA) as indicated in the figure legends. Data are shown as the means ± standard errors of the mean (SEM) if not indicated otherwise. Levels of significance were indicated as follows: ns = non-significant, *p* = 0.05 (*), *p* = 0.01 (**), *p* = 0.001 (***).

**Figure 3:**
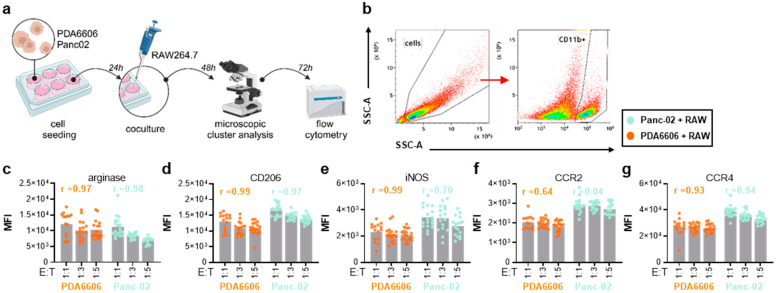
Surface marker profiling of pancreatic cancer-associated macrophages in vitro. (**a**) Scheme of the experimental workflow; (**b**) flow cytometry gating strategy; (**c**–**g**) surface expression of arginase (**c**), CD206 (**d**), iNOS (**e**), C–C chemokine receptor type (CCR) 2 (**f**) and CCR4 (**g**) on cocultured macrophages at different effector-to-target (ET) ratios as determined using multicolor flow cytometry (n = 3). Note: ns = non-significant, MFI = mean fluorescence intensity.

## 3. Results

### 3.1. RAW264.7 Experimental Polarization and Chemokine Release

This work used murine RAW264.7 cells as a macrophage model to further study the interaction with pancreatic cancer cells and the secretion profiles of multiple chemokines potentially involved in this process. To this end, we first differentiated RAW264.7 cells using M-CSF and M1- or M2-inducing agents to confirm the successful differentiation of macrophages (Figure 1a). During the process of macrophage differentiation and activation, cells typically enlarge, leading to distinctly different scatter profiles that can be analyzed using flow cytometry (Figure 1b). In particular, M1 macrophages showed markedly elevated side-scatter values compared to non-stimulated RAW264.7 cells (Figure 1c). M1 and M2 macrophages are known to have altered surface expression profiles representative of hallmark markers, such as inducible nitric oxide synthase (iNOS) and CD11b for M1 macrophages and arginase as a marker of M2 macrophages [15,16]. Accordingly, our experiments showed significantly increased expression levels for the respective molecules (Figure 1c–e), confirming the suitability of RAW264.7 cells to be primed to either macrophage population. Since our work focused on chemokines in the context of pancreatic cancer cells driving macrophage polarization levels, we also investigated the relative surface marker expression of two chemokine receptors, CCR2 and CCR4. Compared to M0 macrophages, the former was significantly decreased in M1 but not M2 macrophages. At the same time, the latter was increased considerably in M1 and decreased in M2 macrophages (Figure 1f).

**Figure 4:**
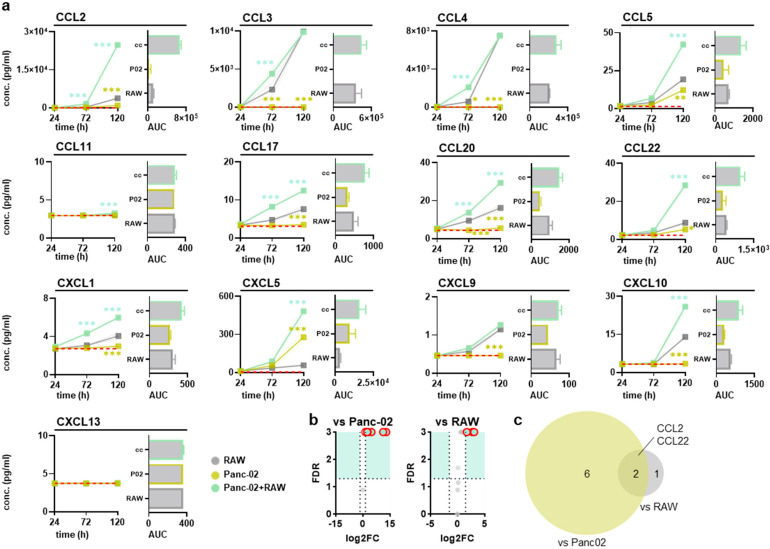
Chemokine release profiles in supernatants of Panc02-conditioned macrophages in vitro. (**a**) Quantified chemokine levels at 24 h, 72 h, and 120 h after coculturing with Panc02 murine pancreatic cancer cells and their calculated areas under the curve (AUC), with the dashed red line indicating the lower detection limit (n = 3); (**b**) volcano plots with false discovery rates (FDR) and critical data points circled in red; (**c**) Venn diagrams showing the overlap of significantly altered chemokine expression levels in the coculture compared to the Panc02 pancreatic cancer cell or RAW264.7 macrophage monoculture. Statistical analysis was performed using two-way analysis of variance (ANOVA) (* *p* < 0.05, ** *p* < 0.01, ***p < 0.001). Note: conc. = concentration, CCL = C–C motif chemokine ligand, CXCL = C–X–C motif chemokine ligand.

To next assess how macrophage polarization changed the chemokine secretion profiles, supernatants of M0, M1, and M2 macrophages were collected and analyzed for 13 different chemokines in parallel (Figure 2a). To achieve a better understanding of the relative changes, the data from 72 h were normalized to the timepoint of adding or not adding reagents for M1 or M2 macrophage polarization at 24 h, at which point the cells had been in M-CSF already for 24 h. When comparing relative increases by calculating the area under the curve (AUC) of these normalized data, a range of chemokines was found to be differentially secreted into the surrounding cell culture medium within these 48 h by M0-, M1-, and M2-like macrophages. When performing statistical analysis against the release trends by M0 macrophages, a range of chemokines was significantly differentially released by M1 macrophages, including CCL2, CCL3, CCL4, CCL5, CCL17, CCL22, CXCL9, and CXCL10 (Figure 2b). For M2 macrophages, several chemokines (CCL5, CCL22, and CXCL10) were also released differentially. This suggested markedly different chemokine secretion patterns by stimulated M1 macrophages. For M2 macrophages, compared to M0 macrophages, only the significantly decreased levels of CCL5, CCL22, and CXCL10 were observed, which also matched the M1 macrophage behavior.

### 3.2. RAW264.7 and Pancreatic Cancer Cocultures Modified Surface Markers and Chemokine Release

**Figure 5:**
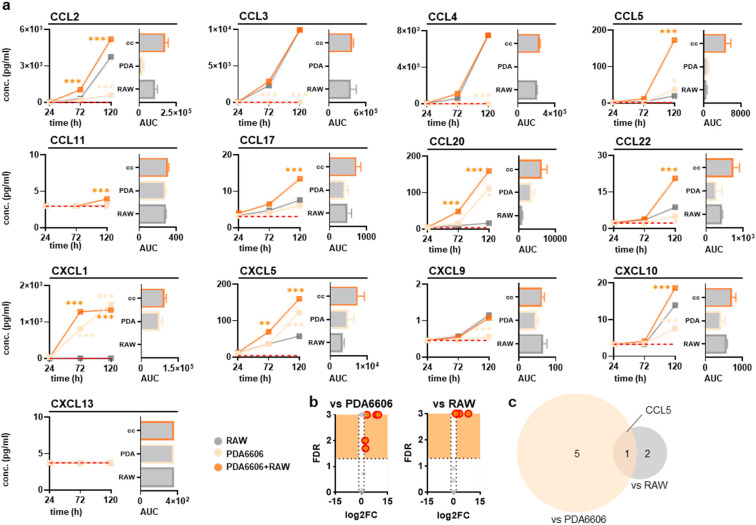
Chemokine release profiles in supernatants of PDA6606-conditioned macrophages in vitro. (**a**) Quantified chemokine levels at 24 h, 72 h, and 120 h after coculturing with PDA6606 murine pancreatic cancer cells and their calculated areas under the curve (AUC), with the dashed red line indicating the lower detection limit (n = 3); (**b**) volcano plots with false discovery rates (FDR) and red circles indicating critical data points; (**c**) Venn diagrams showing the overlap of significantly altered chemokine expression levels in the coculture compared to the PDA6606 pancreatic cancer cell or RAW264.7 macrophage monoculture. Statistical analysis was performed using two-way analysis of variance (ANOVA) (* *p* < 0.05, ** *p* < 0.01, *** *p* < 0.001). Note: conc. = concentration, CCL = C–C motif chemokine ligand, CXCL = C–X–C motif chemokine ligand.

**Figure 6:**
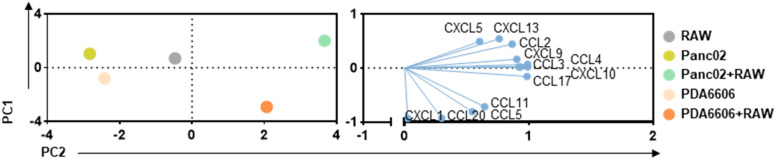
PCA of macrophage and pancreatic cancer cell secretion profiles. Principal component analysis (PCA) of secretion profiles of 13 different chemokines in murine RAW264.7 macrophages, murine Panc02, and murine PDA6606 monocultured cells, or cocultures of macrophages with pancreatic cancer cells, showing PC scores and loadings (n = 3).

To next identify the impact of pancreatic cancer cells on RAW264.7 cells, we used two different murine pancreatic cancer cell lines, PDA6606 and Panc02, for coculture experiments and cellular analysis (Figure 3a). RAW264.7 cells were gated by multicolor flow cytometry by their strong expression of CD11b (Figure 3b), which allowed this subpopulation to be analyzed for further cell surface markers related to macrophage activation and differentiation. Testing the cocultures at different effector-to-target (ET; RAW264.7 macrophages: pancreatic cancer cells) ratios, both cocultures showed a consistent increase in arginase on RAW264.7 cells with increasing numbers of pancreatic cancer cells independent of the tumor cell line investigated (Figure 3c). Similar results were observed for mannose receptor type 1 (CD206) (Figure 3d), pointing to a possibly M2-skewed phenotype in the RAW264.7 cells after coculturing with pancreatic cancer cells. Interestingly, there was also a slight trend toward an increase in iNOS with PDA6606, which was even more pronounced with Panc02 cocultures (Figure 3e). Since we were also interested in the surface marker expression profiles of chemokine receptors CCR2 and CCR4, the expression of both was also measured using multicolor flow cytometry and found to associate moderately (Figure 3f) and strongly (Figure 3g) with pancreatic cancer ET ratios for CCR2 and CCR4, respectively. These results suggested an altered RAW264.7 phenotype due to cocultures and surprisingly similar results for the two cell lines used, PDA6606 and Panc02. Next, the chemokine release profiles in the supernatants of monocultures and cocultures were analyzed for both cancer cell lines. When statistically compared to the levels in RAW264.7 alone, coculturing with Panc02 showed markedly increased (i.e., at least 2-fold and significantly changed) levels of CCL2, CCL5, CCL22, and CXCL5 at 120 h of incubation (Figure 4a). Except for CXCL13, none of the tested cytokines showed a significantly increased release at either 72 h or 120 h of incubation in cocultures compared to RAW264.7 monocultures. When running an alternative statistical analysis plotting log_2_ fold changes compared to false discovery rates (Figure 4b), the two chemokines uniquely increased in cocultures against monocultures were CCL2 and CCL22 (Figure 4c). Principally similar results were seen with PDA6606 cocultures, while in this case, CCL3, CCL4, CXCL9, and CXCL13 did not show any significant changes in cocultures compared to RAW264.7 monocultures at either 72 h or 120 h of incubation (Figure 5a). Here, markedly increased chemokines were CCL5, CCL20, CCL22, CXCL1, and CXCL5, again showing a substantial overlap with results seen with Panc02 cells. Volcano plots (Figure 5b) revealed CCL5 to be the chemokine uniquely modulated when comparing against both RAW264.7 and PDA6606 cocultures (Figure 5c). Principal component analysis (PCA) confirmed a principal similarity of chemokine release profiles between pancreatic cancer monocultures (left) and cocultures with RAW264.7 cells (right) (Figure 6). The loading plots further indicate those chemokines that are critical in annotating values of principal components 1 and 2 (PC1, PC2).

## 4. Discussion

Macrophage polarization is a critical trait in pancreatic cancer TME and is associated with poor treatment outcomes and survival [17]. Understanding the intricate interplay between pancreatic cancer cells and their ways of modulating macrophage phenotypes is essential for understanding potential interventional approaches to improve the existing therapies [18]. With the intention of focusing on chemokines, we aimed here to provide clues regarding a straightforward in vitro coculture model that informs on chemokines potentially critical in shaping the TME and is involved in pancreatic cancer and macrophage interplay.

For decades, chemokines have been studied to analyze cellular polarization among immune and nonimmune cell types in many diseases and processes, such as angiogenesis [19,20]. Accordingly, chemokines have received increasing attention in the biology of several cancer types, including lung cancer, head and neck cancer, and pancreatic cancer [21,22,23]. However, little is known about the chemokine profiles released upon macrophage and pancreatic cancer monocultures and cocultures, which motivated our current study. In a previous report, PCR profiling was performed in LA-4 alveolar lung macrophages, and upon LPS stimulation, a marked increase in, for instance, CCL2, CCL7, CCL17, and CXCL10 was observed [24], similar to our data. The cytokine release of human THP-1 monocyte-derived macrophages was also observed for, e.g., CCL2 through gene expression analysis and ELISA [25]. Interestingly, in subcutaneously induced murine Panc02 tumors, the most potent expression was found for chemokines CCL2, CCL7, and CCL22 in vivo [26].

Our analysis of CCR2 and CCR4 revealed their differential modulation upon macrophage polarization and pancreatic cancer cell coculture. CCR2, a critical receptor of CCL2 [27], was initially identified as a crucial receptor for cytotoxic T cell migration towards cancer cells [28]. Interestingly, the CCR2–CCL2 axis has also been deemed essential in tumor killing, metastasis, and immune control, including in the context of macrophages [29,30,31,32,33]. In pancreatic cancer, it has been shown that tumor cells begin secreting CCL2 soon after malignant transformation, acting as a potent chemoattractant for monocytes and macrophages [34]. In human pancreatic ductal adenocarcinoma, the CCL2–CCR2 chemokine signaling axis has been identified as having prognostic importance [35]. Targeting this signaling axis via CCR2 blockade or CCL2-neutralizing antibodies has been shown to slow tumor progression and shunt TAMs from the TME in several preclinical tumor models, including pancreatic cancer in vivo [35,36,37]. CCR4 is similarly important regarding infiltrating immune cells in the TME. In a previous study, we showed in a syngeneic in vivo model using PDA6606 cells in C57BL/6 mice that pharmacological or steric inhibition with blocking antibodies targeting CCR4 significantly increased the survival of tumor-bearing mice [38]. The same study achieved similar results using CCR4 knockout mice, which showed substantially fewer immune cell infiltrates, including macrophages. Although the inverse correlation of surface marker expression levels with ET ratios might, in general, be influenced by confounding factors such as nutrient competition or stress in high-density cocultures, our data are, therefore, in line with the previous findings of macrophage polarization in the context of pancreatic cancer in vitro and in vivo. The ligands of CCR4, also known as CD194, are CCL17 and CCL22 [39], both known to facilitate the infiltration of immunosuppressive regulatory T cells into the microenvironment [40], with CCL22 also being significantly increased in M2-polarized RAW264.7 macrophages in this study. During the cocultures in our study, the CCL17 and CCL22 levels were significantly enhanced compared to macrophages alone. CCL22 has been identified as a murine and human M2 macrophage marker [11]. This supports the idea that our presented coculture model recapitulates macrophage features phenotypically observed in cancer and would allow us to study chemokine involvement further.

Most CCL and CXCL chemokines were previously demonstrated to play critical roles in M1 and M2 macrophage polarization [41]. In our study, the candidates stood out as being exceptionally strongly regulated in the cocultures over the pancreatic cancer and RAW264.7 monocultures, namely the previously discussed CCL2 and CCL22 for Panc02 cells and CCL5 for PDA6606 cells. CCL5, also called RANTES, plays multifaceted roles in cancer. It has been associated with promoting antitumor immunity via dendritic cells [42] and was previously associated with both M1 and M2 macrophage polarization [43], despite our macrophage polarization data suggesting elevated levels with M0 compared to M1 and M2 macrophages. Vice versa, previous studies could show that the secretion of CCL5 along with TNF-α by macrophages induced acinar-to-ductal metaplasia in pancreatitis, pointing to their role in early malignant transformation [44]. In mice, elevated levels of CCL5 have further been shown to promote tumor immune evasion and mediate mesenchymal stromal cells homing into the TME, enhancing tumor growth [45,46]. Along similar lines, a retrospective analysis of transcriptomic data obtained from patient-derived pancreatic cancer samples outlined a positive correlation between high expression levels of CCL5 and poor overall survival [47]. Notably, in the context of chronic inflammation and outside of oncology, CCL5 is associated with M1 rather than M2 macrophages [48]. In this regard, it is generally seen as a proinflammatory chemokine originally discovered in T cell biology, and is also readily produced by monocytes [49] that are activated in a receptor-dependent fashion (CCR1, CCR3, CCR5) at low RANTES levels and in a receptor-independent fashion at high RANTES concentrations where it acts as a mitogen [50]. In the TME, various tumors and TAMs are believed to release CCL5 to attract additional monocytes and macrophages into the TME [51]. This ultimately amplifies the army of immunosuppressive cells and fuels tumor growth, especially since several chemokines are capable of attracting myeloid cells into the TME [52].

This study provides a descriptive characterization of chemokine signatures to elucidate the consequences of the tumor–macrophage interaction in the context of pancreatic cancer. Notably, RAW264.7 surface marker and chemokine patterns were comparable with those of two pancreatic cancer cell lines, outlining the general suitability of our model. Previous studies demonstrated good reproducibility of the behavior of primary macrophages in side-by-side comparisons with RAW264.7 macrophages [44,53,54,55]. As such, the RAW264.7 macrophage cell line has been used in studies, e.g., to investigate chemotherapy-induced immunosuppression [56] or to evaluate immunochemotherapy targeting TAMs [57] or the influence of tumor exosomes on macrophage polarization [58]. However, validation of our results using, e.g., monocyte-derived human macrophages, syngeneic tumor models in immunocompetent mice, along with histochemical analysis of patient-derived tumor tissues, would be needed to strengthen the clinical relevance of our results. Along similar lines, follow-up studies should clarify the functional consequences and translational relevance of identified targets for macrophage behavior. This would not only be of scientific interest in tumor immunology, but might also help overcome the current limitations of immunotherapies in treating pancreatic cancer.

## 5. Conclusions

Our in vitro model demonstrated that it is suitable for performing cocultures of murine macrophages and murine pancreatic cancer cells to identify functional features of such an interaction. Intriguingly, two pancreatic cancer cell lines provided similar results regarding chemokine release and surface marker profile alterations in macrophages, underlining the significant themes emphasized by our and previous data in macrophage polarization of pancreatic cancer. Future studies may extend these findings to human and primary cells, including functional testing in more complex three-dimensional cultures and in vivo models. In addition, functional studies using receptor-blocking antibodies and genetic manipulation may shed additional light on the roles of individual chemokines in macrophage polarization.

## Data Availability

The underlying data of this manuscript are available from the corresponding author upon reasonable request.
